# Park use, perceived park proximity, and neighborhood characteristics: Evidence from 11 cities in Latin America

**DOI:** 10.1016/j.cities.2020.102817

**Published:** 2020-10

**Authors:** Mika R. Moran, Daniel A. Rodríguez, Andrea Cotinez-O'Ryan, J. Jaime Miranda

**Affiliations:** a Institute for Urban and Regional Development, University of California Berkeley, Berkeley, CA 94720, USA; b Department of City and Regional Planning, University of California Berkeley, Berkeley, CA 94720, USA; c Department of Public Health, Pontificia Universidad Católica de Chile, Santiago, Chile; d Departamento de Educación Física, Deportes y Recreación, Universidad de la Frontera, Temuco, Chile; e Department of Medicine, School of Medicine, Universidad Peruana Cayetano Heredia, Lima, Peru; f CRONICAS Centre of Excellence in Chronic Diseases, Universidad Peruana Cayetano Heredia, Lima, Peru

**Keywords:** Park use, Perceived park proximity, Built environment, Perceived social disorder, Latin America

## Abstract

This study examines how park use may be associated with perceived park proximity, neighborhood-built environment and perceived social disorder in Latin American cities. The study uses self-reported data from the 2016 CAF survey, including 7,970 urban residents from 11 cities across Latin America. Results show positive graded associations between perceived park proximity and use, holding all others constant. Additional factors that were found to be associated with park use are neighborhood formality and related built-environment characteristics, including paved streets and sidewalks. Park use was mostly unrelated to perceived social disorder, with the exception of indigence, with which it is was positively associated. Stronger associations between park proximity and use were observed among those who reported higher prevalence of indigence or begging in their household block. These findings stress the importance of perceived park proximity in enhancing their use in urban Latin America, and challenge the role of social disorder and crime as a barrier for park use.

## Highlights

• Park proximity is associated with park use in a dose-response relationship.
• Park use is less prevalent in informal compared to formal neighborhoods.
• Park use is more prevalent in areas with paved streets and sidewalks.
• Park use is mostly unrelated to neighborhood social disorder conditions.
• Park use increases with the combined effects of indigence and high park proximity.

## 1 Introduction

Parks and green open spaces are essential for public health due to their associations with various physical-, social- and mental-health benefits (Bedimo-Rung, Mowen, & Cohen, 2005; de Blasio, 2016; Evenson, Wen, Hillier, & Cohen, 2013; Kaczynski & Henderson, 2007; Markevych et al., 2017; Sarkar, Webster, & Gallacher, 2018; van den Bosch & Sang, 2017). Parks provide opportunities for health-enhancing physical activity and social interactions, which may consequently contribute to community social capital (Bedimo-Rung et al., 2005; Markevych et al., 2017). Park use is important for public health as it may involve physical activity in parks as well as while travelling to and from parks (i.e., walking, biking) (Evenson, Wen, Golinelli, Rodríguez, & Cohen, 2013; Evenson, Wen, Hillier, & Cohen, 2013). Overall, park use was found to be associated with higher levels of physical activity (Leslie, Cerin, & Kremer, 2010) and related health benefits (Mowen, Orsega-Smith, Payne, Ainsworth, & Godbey, 2007).

Motivated by these benefits, local governments and agencies have sought to increase the availability of parks for local populations as a useful strategy to enhance park use (Kaczynski & Henderson, 2007) and related health benefits (Markevych et al., 2017; Mowen et al., 2007). These efforts are often guided by socio-ecological models (Sallis et al., 2016; Sreetheran & Van Den Bosch, 2014), according to which human behavior has multiple levels of influences, ranging from environmental conditions (e.g., parks attributes and their surroundings), through social factors (e.g., personal safety and social disorder), to individual's characteristics (e.g., sociodemographic characteristics, attitudes and preferences).

Previous studies suggest that park use increases with increasing proximity to parks (Dunton, Almanza, Jerrett, Wolch, & Pentz, 2014; Kaczynski & Henderson, 2007; Kaczynski, Potwarka, Smale, & Havitz, 2009), although mixed evidence also exists (Lachowycz & Jones, 2011). Perceived park proximity was also found to be related to park use (Kaczynski & Henderson, 2007), in some cases even after accounting for the actual measured distance (Leslie et al., 2010). Overall, people who live near green spaces are more likely to engage in health-enhancing physical activity (Bedimo-Rung et al., 2005; Kaczynski & Henderson, 2007).

In addition to park proximity, the physical environment within and surrounding parks is likely to enhance park use and related health-benefits. Parks are more likely to be used if they are large, have high quality and well-maintained infrastructure, contain supportive facilities and amenities (e.g., trails, ball courts, and rest areas) and offer supervised activities (Cohen et al., 2016; McCormack et al., 2004; Wendel, Zarger, & Mihelcic, 2012). Neighborhood features may also impact park use, but, unlike internal park features, neighborhood influences on park use have been less studied and empirical evidence is inconclusive (Kaczynski, Johnson, & Saelens, 2010; Koohsari, Karakiewicz, & Kaczynski, 2013; Shores & West, 2010; Van Dyck et al., 2013). To illustrate, a study from the USA and Belgium showed that parks are more likely to attract users if located in highly walkable neighborhoods, characterized by high values of residential density, street connectivity and land use mix (Van Dyck et al., 2013). However, contrary findings from Australia have shown that walking to and within public open spaces was less common among people living in highly connected streets, compared to those living in cul-de-sac areas (Koohsari et al., 2013). Similarly, in Canada, land use mix nearby parks was found to be related with decreased physical activity within those parks (Kaczynski et al., 2010). Another study from the USA showed that park visits in urban areas were more frequent but less active than those in rural areas (Shores & West, 2010).

Additional influences on park use involve the neighborhood social environment (Markevych et al., 2017) and personal safety (Cohen et al., 2016; Sreetheran & Van Den Bosch, 2014). Parks located in high crime neighborhoods are less likely to be utilized for physical activity and social interactions, but are rather more likely to accommodate social disorder, which, in turn, may increase fear and park avoidance (Bedimo-Rung et al., 2005). The observed associations between social disorder and park use may vary depending on the specific facet of social disorder under investigation (Bogar & Beyer, 2016; Cohen, Han, Derose, et al., 2016; Han, Cohen, Derose, Li, & Williamson, 2018). For example, two recent studies from low income neighborhoods in Los Angeles (California, USA) yielded contrary results concerning the crime-park use associations: Han et al. (2018) found that gun violence is associated with less park use, while Cohen, Han, Derose, et al. (2016) found that the presence of gangs and intimidating groups in parks is associated with increased park use. These contradicting results can be explained by the nature of each social disorder outcome. Given its significant threat to public health and safety, people may be more likely to respond to gun violence by avoiding the outdoors. However, the mere presence of gangs, despite being intimidating, may have a lesser effect on individual behavior. Furthermore, gangs may be drawn to parks in central locations that may also attract normative park users (e.g., joggers, pedestrian commuters). Despite the latter, it is noteworthy that the majority of the literature to date suggest that neighborhood crime is associated with less park use (Kuo & Sullivan, 2001; Sreetheran & Van Den Bosch, 2014). Furthermore, reducing neighborhood crime, especially through providing more recreational destinations nearby, has been suggested as an effective obesity prevention strategy through its positive influence on physical activity in those destinations (Powell-Wiley et al., 2017).

On top of environmental and social factors, the way in which individuals perceive and interact with their local environment is also likely to impact park use. Park visits are likely to be more common among individuals who perceived their local environment as safe in terms of both traffic (Parra et al., 2010) and crime (Leslie et al., 2010). However, individuals who have previous experience as crime victims and/or have prior information about crime incidents in their local environment may be more likely to avoid visiting parks out of fear (Sreetheran & Van Den Bosch, 2014). Fear of crime and related park avoidance might be more common among certain population groups, such as women, the elderly, minorities and low-income groups (Bedimo-Rung et al., 2005; McCormack et al., 2004).

While evidence on parks and related health benefits is abundant, the majority come from the Global North (Sallis et al., 2016; Sreetheran & Van Den Bosch, 2014), leading to an underrepresentation of areas where urban green spaces are especially important considering the rapid growth and related environmental and health challenges faced by local residents. With few exceptions (Gomez et al., 2010; Hallal et al., 2010; Jáuregui et al., 2016), Latin America is a less studied region with heightened vulnerability due to its level of urbanization and high social inequalities among and within cities (Vereinte Nationen, 2013), a deficit and unequal distribution of urban green space (Rigolon, Browning, Lee, & Shin, 2018), and high crime rates (Sreetheran & Van Den Bosch, 2014).

Despite their paucity, studies from Latin America overall support the majority of evidence from the Global North showing positive associations between park characteristics (e.g., high proximity, size, amenities), park use and related health benefits (Fermino, Reis, Hallal, & Kaczynski, 2015; Mena et al., 2015, Mena et al., 2016; Parra et al., 2010; Salvo et al., 2017; Scopelliti et al., 2016; Wendel et al., 2012). While research interest on parks in Latin America is increasing, studies to date mostly focused on park use associations with health-related outcomes (Fermino et al., 2015; Parra et al., 2010) and/or with park characteristics (Krellenberg, Welz, & Reyes-Päcke, 2014; Scopelliti et al., 2016; Wendel et al., 2012). The current study adds to this emerging literature by exploring the potential park use in different neighborhood conditions, including the comparison between formal and informal neighborhoods. Such exploration is especially important in Latin America given the profound diversities within and across cities, and especially between formal and informal neighborhoods, which greatly contribute to intra-urban inequalities (Caprirolo et al., 2017).

In this study, we examine the direct and moderating associations of perceived park proximity and neighborhood characteristics with park use in a sample of residents from 11 Latin American cities through the following questions: (1) Is perceived park proximity associated with park use? (2) Is park use associated with neighborhood-built-environment and perceived social disorder? (3) Do the associations between perceived park proximity and park use vary by neighborhood-built environment and perceived social disorder? In line with our research questions, we hypothesized that (1) Perceived park proximity is positively associated with park use; (2.1) supportive built environments are positively associated with park use, (2.2.) perceived social disorder is negatively associated with park use; and (3) the associations between perceived park proximity and use are enhanced in neighborhoods with supportive built-environments, and attenuated in neighborhoods with high perceived social disorder. We based the third hypothesis on the socio-ecological theory, according to which, a combination of favorable built- and social-environment conditions are presumed to enhance desired behaviors, such park use (Sallis et al., 2016; Sreetheran & Van Den Bosch, 2014). We also base our hypotheses on the results of the abovementioned studies linking park use with higher levels of park proximity (e.g., Gomez et al., 2010; Kaczynski & Henderson, 2007), built-environment walkability (Van Dyck et al., 2013) and personal safety (Kuo and Sullivan, 2001).

## 2 Materials and methods

### 2.1 Sample and procedures

This study uses a cross-sectional, stratified, representative survey held by the Development Bank of Latin America (CAF or *Corporación Andina de Fomento*, henceforth: CAF survey) during November 2016 through January 2017. The sampling plan and questionnaire are described elsewhere (*Cuestionario ECAF 2016.pdf*, n.d.; Development Bank of Latin America, 2017). Respondents were heads of household or, in the absence of the head a household, an adult household residents aged 20–60. The survey includes responses from 12,905 households in the following 11 Latin American cities: La Paz (Bolivia), Lima (Peru), Mexico City (Mexico), Montevideo (Uruguay), Panama City (Panama), Quito (Ecuador), Sao Paulo (Brazil), Buenos Aires (Argentina), Bogota (Colombia), Caracas (Venezuela) and Fortaleza (Brazil). The sample from the latter four cities was stratified by neighborhood formality/informality, defined as a set of more than 50 contiguous dwellings with the following characteristics: (1) no property title, (2) building deficiencies, and (3) lack of formal access to utilities such as water, electricity and sanitation.

### 2.2 Measures and variables

This study includes one outcome – park use, and three main predictors – perceived park proximity, neighborhood-built environment and perceived social disorder.

#### 2.2.1 Park use

This was a binary variable based on the single ‘yes/no’ response to the question: “Do you or another member of your household use parks, squares or green areas on a regular basis?”

#### 2.2.2 Perceived park proximity

Respondents were asked to describe the time it would take them or other household members to walk to the nearest “park, square or green space” by selecting one of three options: “less than 10 min”, “10–30 min” and “more than 30 min”. Using the category “more than 30 min” as a reference group, the following two dummy variables were created: *high park proximity* – having a park in less than 10 min walking from home, and *reasonable park proximity* – having a park in 10–30 min walking from home.

#### 2.2.3 Perceived neighborhood social disorder

Respondents were asked how often each of the following social disorder conditions occurred in their household’s block: *gangs, prostitution, indigence or begging* and *assault and/or crime*. To answer this question, respondents were presented with a scale of 1–5 when: 1 = “never”, 2 = “rarely”, 3 = “sometimes”, 4 = “almost always”, 5 = “always”. To ease comparability with drug-use, these answers were then recoded to create a binary variable when 0 = "never or rarely" and 1 = “at least sometimes”. Each social-disorder condition was analyzed separately due to their different nature, which may shape their impact on park use. *Drug use* activity was also assessed though slightly different, by a single question asking respondents whether “drug dealing or drug use occur within three blocks or less” from their home (0=no, 1=yes).

#### 2.2.4 Neighborhood built environment

Four built environment characteristics were included, some of which were self-reported by respondents (perceived street lighting, perceived proximity to destinations), while others were documented by the surveyors during data collection (paved streets, sidewalks).

*Perceived street lighting* – respondents reported whether or not they had “poorly lit street” within three blocks from their home. For analysis purposes, the respondents' answers were inverted to create a variable representing proper street light (1 = “yes”, 0 = “no”).

*Sidewalks* – surveyors reported whether respondents had sidewalks in the street where they live or not.

*Paved street* – surveyors reported the type of the street where respondent's live by selecting one of four categories: “paved”, “alley”, “dirt” or “other”. For analysis purposes, this was recoded as 1 = “paved street” and 0 = “non-paved street”.

*Perceived proximity to destinations* – this variable was defined as having high proximity (within 10 min walking distance from home) to at least three of the following destinations: Public facility (public library, cultural center), community/sports/recreation center, school, daycare, hospital and police station. Respondents were asked to describe the time it would take them or other household members to walk to each of the aforementioned destinations by selecting one of three options: “less than 10 min”, “10–30 min” and “more than 30 min”. Responses were recoded into: 1 = “less than 10 min” 0 = “longer than 10 min” and added up to yield a composite variable of *perceived proximity to destinations* with values ranging from 0 to 6, where 0 = “none of the destinations is within 10 min walking distance from home”, 6 = “all six destinations are within 10 min walking distance from home”. For analysis purposes, this variable was recoded using the median value of the frequency distribution of responses (M = 3) as a cutoff, where: 0 = “two destinations or less are within walking distance from home” (henceforth: *low proximity to destinations*), and 1 = “three destinations or more are within walking distance from home” (henceforth: *high proximity to destinations*).

In addition to the above, neighborhood formality (used in the sampling process for four cities) was also included in the analysis.

#### 2.2.5 Individual-level demographic and socioeconomic characteristics

Self-reported demographic characteristics included: sex, age, having school aged children (aged 4–18), length of neighborhood residency (in years), and number of persons living in the household. Socioeconomic indicators included automobile ownership, employment, and education. Self-rated health was examined through a single question asking respondents to assess their health using one of the following three options: “*good*”, “*regular*”, “*poor*”. This self-rated health single question variable refers mostly to physical health (Manderbacka, 1998), and was found to be associated with various health outcomes (Bowling, 2005).

### 2.3 Statistical analysis

Stata v15 (Stata Corporation, College Station, TX, USA) was used for statistical analysis. Conventional descriptive statistics were employed to describe the sample and variables. Multilevel logistic regression models were used to predict park use, including individual-level independent variables and a random intercept at the city level to account for heterogeneity across cities. Sampling weights were used in the CAF survey, but the probability of selection at each level was unavailable and hence sampling weights were not used in this analysis. First, bivariate-associations between park use and each independent variable of interest (park proximity, built environment, social disorder) were assessed, while adjusting for individual socio-demographic variables. We then examined combined influences by entering all variables in a multivariate model while incrementally adding three blocks of variables: The first model included perceived park proximity and built environment variables and the second model included the first one plus perceived social disorder variables. These two models help answer the first and second research questions. The third model addresses the third question in our research by including the potential addition of interaction terms between perceived park proximity with perceived neighborhood-built-environment and social disorder variables (which were found to be significant in the first two models). Estimates are accompanied by 95% CIs and conventional level of *p* ≤ .05 was taken to represent statistical significance when interpreting model results. In order to assess potential bias due to residential self-selection, we conducted a sensitivity analysis by excluding from the sample 43 respondents who reported “proximity to parks and squares” as one of the main reasons for choosing their neighborhood. The results remained almost unchanged, suggesting that residential self-selection is not likely to affect our analysis.

## 3 Results

### 3.1 Sample characteristics

Of the 12,905 respondents of the CAF survey, 7,970 had complete information on park use, neighborhood characteristics and socio-demographic characteristics. 4,935 individuals were excluded from the original sample because they did not have complete information on park use, 446 did not have complete information on neighborhood-built environment, 961 did not have complete data on neighborhood social disorder, and 3,528 did not have all individual socio-demographic characteristics. The estimation sample used for this analysis (*n* = 7,970) did not differ from the original CAF survey sample (*N* = 12,905) with respect to sociodemographic and socioeconomic characteristics.

Table 1 presents the sample characteristics in the total sample and by park use (Appendix 1 presents descriptive statistics by city). 63% of the study sample reported using parks on a regular basis (Table 1). More than half of the sample (55%) reported having high proximity to parks (less than 10 min walking from home), and only 14% reported having low proximity to parks (more than 30 min). 81% of respondents reside in formal and the remaining 19% in informal neighborhoods. Most of respondents reside in paved streets (79%) with sidewalks (70%), and about half of the sample reported having streetlights (52%) and high proximity to destinations (49%). The most commonly reported condition of social disorder was drug use, reported by 58% of the sample, followed by assault or crime (50%), indigence or begging (45%), gangs (44%) and prostitution (18%). In terms of sociodemographic characteristics, 59% of the respondents are female, 63% were employed, 30% own an automobile (one or more), and 52% had a high-school education or higher. The majority of respondents rated their own health as good (64%), only 4% as bad and the remaining 32% as regular health. The average age of respondents was 40, and they lived in their neighborhood, on average, for 20 years.

**Table 1:** Descriptive statistics of the study sample and by park use (n = 7,970)

Variables	Total sample (count, percent)	Park users (percent)	Non-park users (percent)
Park users (ref: Non park users)	5,021 (100%)	100%	0%
*Park proximity*			
- More than 30 minutes' walk	1,120 (14.05%)	39.82%	60.18%
- 10-30 minutes' walk	2,482 (31.14%)	60.03%	39.97%
- Less than 10 minutes' walk	4,368 (54.81%)	70.63%	29.37%
*Neighborhood characteristics*			
Formal neighborhood	6,417 (80.51%)	66.46%	33.54%
Informal neighborhoods	1,553 (19.49%)	48.68%	51.32%
*Built environment*			
Paved street within block (ref: other)	6,269 (78.66%)	63.81%	36.19%
Sidewalks within block (ref: no)	5,596 (70.21%)	64.11%	35.89%
Streetlights within three blocks (ref: no)	4,118 (51.67%)	64.10%	35.90%
High proximity to destinations (ref: no)	3,878 (48.66%)	63.69%	36.31%
*Social disorder*			
Drug use	4,609 (57.83%)	61.55%	38.45%
Gangs	3,497 (44.24%)	63.68%	36.32%
Prostitution	1,455 (18.26%)	58.00%	42.00%
Indigence or begging	3,572 (44.82%)	61.87%	38.13%
Assault or crime	3,981 (49.95%)	61·92%	38·08%
*Individual characteristics*			
Male	3,293 (41.32%)	64.99%	35.01%
Female	4,677 (58.68%)	62.65%	37.35%
Age⁎	40.02 (11.09)	38.96 (10.81)	41.78 (11.33)
Length of neighborhood residency⁎	20.32 (14.87)	19.20 (14.48)	22.28 (15.33)
Having school aged children (ref: no)	5,181 (65.00%)	66.30%	33.70%
Automobile owner (ref: no)	2,395 (30.05%)	67.31%	32.69%
Employed (ref: unemployed)	5,000 (62.74%)	65.36%	34.64%
Num of person in the household⁎	4.27 (1.70)	4.32 (1.67)	4.18 (1.73)
*Education*			
- Less than high school	3,812 (48.00%)	44.93%	53.27%
- High school or higher	4,130 (52.00%)	55.07%	46.37%
*Self-rate health*			
- Bad	302 (3.79%)	48.34%	51.66%
- Regular	2,574 (32.30%)	59.87%	40.13%
- Good	5,094 (63.91%)	65.96%	34.04%
*City of residence*			
Buenos Aires	1,034 (12.97%)	69.92%	30.08%
La Paz	528 (6.62%)	67.23%	32.77%
Sao Paulo	628 (7.88%)	55.57%	44.43%
Fortaleza	938 (11.74%)	35.71%	64.29%
Bogota	1,021 (12.81%)	71.79%	28.21%
Quito	618 (7.75%)	82.85%	17.15%
Lima	663 (8.32%)	72.85%	27.15%
Montevideo	617 (7.74%)	76.74%	23.26%
Caracas	1,043 (13.09%)	44.58%	55.42%
Panama City	318 (3.99%)	53.46%	46.54%
Mexico City	562 (7.08%)	77.58%	22.42%

NA = not applicable.

⁎ Values represent mean and standard deviation (n = 7,970).

### 3.2 Multivariate associations between park use with neighborhood-built environment and perceived social disorder

Appendix 2 presents associations of park use with park proximity, neighborhood characteristics and perceived social disorder, after accounting for individual characteristics. Graded associations were observed between perceived park proximity and park use with the odds of using parks being 3.4 times greater among those living within 10 min and 2.1 times greater among those living within 10–30 min' walk to a park compared to those living more than 30 min' walk. Variables that were found to be borderline significant (*p* < .2) in the adjusted bivariate models (Appendix 2) were included in the multivariate models presented in Table 2. As shown, the graded associations between perceived park proximity and park use remained significant in the multivariate models, but slightly decreased in magnitude after adjusting for other variables (Table 2 models 1 and 2). Park use was associated with some, but not all, built environment attributes. Park use was lower in informal neighborhoods and higher among those living in paved streets and in streets that had sidewalks (Table 2 model 1). Of the perceived social disorder variables, only indigence or begging was found to be associated with park use, and the direction of the association was counter to our hypothesis, showing increased odds of park use in the presence of indigence or begging in the neighborhood block (Table 2 model 2). Finally, the combined effect by perceived park proximity and the perceived presence of indigence or begging was examined (Table 2 model 3). To facilitate interpretability, the original 3-rank variable of perceived park proximity was used in this model for both the main effect and interaction term. Results of this model show that the associations between perceived park proximity and use were stronger among those who reported having indigence or begging near their home. However, after accounting for this interaction, the presence of indigence or begging alone was no longer associated with park use. Interactions between perceived park proximity and built environment characteristics (sidewalks, paved streets, street lights and perceived proximity to destinations) were also examined (to test the third research hypothesis). However, these associations were found to by null (results not reported). We further examined interactions between park proximity with individual characteristics (age, sex, education etc.) and neighborhood formality, but these were also found to have null associations with park use. Appendix 3 presents adjusted associations between perceived park proximity with park use after stratifying the sample by neighborhood formality and sociodemographic characteristics.

**Table 2:** Adjustedⁱ multivariate associations between park proximity and neighborhood characteristics with park use, based on logistic random intercept models (n=7,970)

	Model 1 Perceived park proximity + neighborhood type + BE	Model 2 (1) + perceived social disorder	Model 3 (2) + interaction term
	OR (CI)	OR (CI)	OR (CI)
***Perceived park proximity*** (ref: more than 30 min)			
10-30 minutes' walk	2.05 (1.75-2.39)***	2.04 (1.74-2.39)***	
Less than 10 minutes' walk	3.36 (2.88-3.92)***	3.33 (2.86-3.88)***	
Park proximity (ordinal^a^)			1.65 (1.50-1.82)***
***Neighborhood characteristics***			
***Neighborhood type***			
Informal neighborhood	.64 (.55-.75)***	.66 (.57-.77)***	.65 (.56-.76)***
***Built environment***			
Streetlights within three blocks (self-reports)	.97 (.87-1.08)	.97 (.86-1.08)	.98 (.88-1.10)
Sidewalks	1.21 (1.07-1.36)*	1.16 (1.02-1.32)*	1.16 (1.02-1.32)*
Paved street	1.19 (1.04-1.37)*	1.16 (1.00-1.33)*	1.16 (1.00-1.34)*
High proximity to non-park destinations	1.21 (.88-1.10)	.97 (.87-1.08)	.96 (.86-1.06)
***Perceived social disorder***			
Drug use		.94 (.84-1.06)	
Indigence or begging		1.16 (1.04-1.29)**	.77 (0.54-1.08)
Perceived park Proximity*Perceived social disorder			1.19 (1.03-1.36)*
***Sociodemographic characteristics***			
Age	.98 (.97-.98)***	.98 (.97-.98)***	.98 (.97-.98)***
Female (ref: male)	.92 (.83-1.03)	.92 (.83-1.03)	.92 (.82-1.03)
Having school aged children (ref: no)	1.21 (1.08-1.36)*	1.21 (1.08-1.36)*	1.21 (1.08-1.36)*
Automobile owner (ref: no)	1.01 (.90-1.14)	1.01 (.90-1.14)	1.01 (.89-1.14)
Employed (ref: unemployed)	1.14 (1.02-1.27)*	1.13 (1.01-1.27)*	1.13 (1.01-1.27)*
High school or higher (ref: less than high school)	1.16 (1.04-1.30)*	1.15 (1.03-1.29)*	1.16 (1.04-1.28)*
Self-rated health	1.24 (1.13-1.36)***	1.24 (1.13-1.37)***	1.24 (1.13-1.37)***
Length of neighborhood residency	1.00 (.99-1.00)	.99 (.99-1.00)	..99 (.99-1.00)
Num of person in the household	1.07 (1.04-1.10)***	1.06 (1.04-1.10)***	1.07 (1.04-1.10)***
Constant	.68 (.39-1.20)	.62 (.35-1.11)	.43 (.23-.79)*
Variance of random intercept	.39 (.17-.92)***	.41 (.17-.95)***	.41 (.17-.96)***
Number of observations	7,970	7,970	7,970
Number of groups	11	11	11
AIC	9,101.8	9,096.20	9,092.82

* p≤.05, ** p≤.01, *** p≤.001

Significant values are in bold

a Park proximity coded as: 1=more than 30 min, 2=10-30 min, 3=less than 10 min.

## 4 Discussion

Parks and green spaces are associated with various physical-, social- and mental-health benefits. Abundant studies, mostly from the Global North, have previously examined park use associations with park proximity and neighborhood characteristics; however, considerably less attention has been paid to how these two factors may interact in relation to park use, despite such interactions being intrinsic to socioecological frameworks that guide many studies. We examine associations between perceived park proximity and park use and how these may vary by different perceived neighborhood conditions in a sample of cities in Latin America, an underrepresented world region with high urbanization, considerable greenspace deficit, and high social inequalities. Previous studies in the region are limited in their geographic scope, focusing only on one city and several hundreds of participants. To our knowledge, this is the first multi-city multi-country study on this topic in the region, comprising nearly eight thousand residents from eleven cities in ten countries across central and south America.

The results confirm our hypothesis 1 by showing positive associations between perceived park proximity and park use. While these findings are in line with prior literature (Leslie et al., 2010; Ribeiro, Pires, Carvalho, & Pina, 2015), other evidence also exists. For example, in a Canada, park use was found to be associated with parks' amenities and facilities, but not with park proximity (Kaczynski, Potwarka, & Saelens, 2008). In another Canadian study (Lackey & Kaczynski, 2009), park-based physical activity was found to be null associated with objective and perceived park proximity, but was more likely to occur when objective and perceived proximity were aligned. While considerable evidence from Latin America (Gomez et al., 2010; Hallal et al., 2010; Jáuregui et al., 2016) support our findings on perceived park proximity and use, additional research in this region is needed to understand these associations by using more sophisticated objective and perceived park measures to reflect park features and attributes.

Hypothesis 2.1 is partly satisfied as park use was found to be associated with three of the five built environment variables (neighborhood formality, paved streets and sidewalks). The associations between the built environment and park use can be explained by the fact that supportive neighborhood infrastructure increases park use, on top of the increased proximity to parks. However, in spite of its intuitive appeal, empirical evidence linking park use with the built environment surrounding parks are neither copious nor consistent (Koohsari et al., 2013; Shores & West, 2010; Van Dyck et al., 2013).

Park use was found to be associated with only one of the four perceived social disorder facets, and these associations were in the opposite direction to hypothesis 2.2, linking perceived indigence or begging with increased park use. These findings are surprising given the high-crime rates in Latin America (Caprirolo et al., 2017) along with abundant evidence linking crime with reduced park use (Powell-Wiley et al., 2017; Sreetheran & Van Den Bosch, 2014). Despite this, a few studies yielded results similar to ours. In Porto (Portugal), for example, neighborhood crime (extracted from records) was found mostly unrelated to self-reported leisure time physical activity, with the exception of non-violent crime (theft, verbal offences), which was positively associated with physical activity only among women (Ribeiro et al., 2015). Similarly, in an observational study in low-income neighborhoods in Los Angeles (California) (Cohen, Han, Derose, et al., 2016), more park visitors were documented in parks that had more gangs and intimidating groups in conflict. The underlying processes behind these associations are unclear, but we offer two possible explanations. First, it might be that fear of crime is alleviated in tightly knit communities with strong social networks and support, as those included in the current and aforementioned studies. This explanation is supported by prior study in the US, which found that individual's social integration within their neighborhood community (i.e., knowing neighbors and talking with them often) is related to perceived collective efficacy (i.e., the extent to which neighbors watch out for and help each other), which ultimately may reduce fear of crime (Gibson, Zhao, Lovrich, & Gaffney, 2002). Another possible explanation might be that in areas with extremely high or low crime rates, the influence of crime on park use is attenuated. In this manner, in high-crime urban areas, such as those included in our study and in the one by Cohen, Han, Derose, et al. (2016) crime may be normalized and thus have less effect on people's daily routines. On the other hand, in very safe areas such as Porto (Portugal) (Ribeiro et al., 2015), people may be less aware of or concerned about crime, and thus may be less affected by it. Future research may benefit from further exploring this direction by examining the effects of crime on park use in cities with low, medium and high crime rates.

Our results reject hypothesis 3 that the associations between perceived park proximity and use would be enhanced in neighborhoods with supportive built-environments and attenuated in neighborhoods with high perceived social disorder. Overall, perceived park proximity was found to be associated with more park use in both formal and informal neighborhoods (Appendix 3). These findings suggest that all residents, regardless of socioeconomic strata or neighborhood environment, may use parks more, if those are available nearby. Furthermore, although in informal neighborhoods parks may be fewer and of poorer quality, they may still be frequently used by local populations, who may lack other affordable opportunities for physical activity and recreation. Taken together with the well-established health benefits of park use (Bedimo-Rung et al., 2005; Markevych et al., 2017), our findings point at the potential of increasing park proximity as a means to improve public health and reduce urban health inequalities.

Our results on the interaction between perceived park proximity and indigence or begging (Table 2 model 3) run counter to hypothesis 3, suggesting that increased park use is associated with the combination of having high perceived park proximity and perceived presence of indigence or begging in the neighborhood block. This synergistic effect may be explained by the fact that park users and indigents may both gravitate to parks that share similar traits, such as being in a central location with mixed land uses and vibrant street-life. The latter may alleviate possible fear that may be evoked by indigence or begging. Overall, taken together with the strong, positive and consistent associations between park proximity and park use across most of the cities (see Appendix 4) and in both formal and informal neighborhoods (Appendix 3), our findings stress the importance of parks for local populations in Latin America.

Previous studies point at variations in the associations between social disorder and park use depending on the specific social disorder under investigation (Bogar & Beyer, 2016; Cohen, Han, Derose, et al., 2016; Han et al., 2018). In line with these inconsistencies, our results show positive associations between park use and the presence of indigence or begging, negative (but borderline significant) associations with drug use, and null associations with gangs, prostitution and assault or crime. The positive associations between park use and indigence or begging observed in this study may be attributed to parks in highly dense city centers, which may simultaneously attract indigents and other visitors (e.g., commuters, joggers, brisk walkers). This is in line with previous research linking park use with the presence of intimidating groups (Cohen, Han, Derose, et al., 2016) and with nuisance crime (e.g., criminal mischief, disorderly conduct, narcotics sales and possession, and public drunkenness) (Bogar & Beyer, 2016). However, the null associations with more severe social disorder aspects, such as assault or crime, contradict previous studies (Bogar & Beyer, 2016; Han et al., 2018) and challenge underlying assumptions about social disorder as a barrier to physical activity in the Latin American setting.

Another important aspect to be considered is the unequal distribution of parks across socioeconomic areas. High park proximity and routine park use were less commonly reported by respondents from informal neighborhoods compared to those from formal neighborhoods (results not shown). The low perceived park proximity in informal neighborhoods may reflect actual scarcity of parks in those neighborhoods. However, it may also be that parks are available in informal neighborhoods, but they are in bad condition (e.g., litter, broken benches, graffiti), and thus they are under-reported and under-utilized. This was observed in another recent study from Latin America (Scopelliti et al., 2016), in which lower income groups reported lower perceived accessibility to urban green spaces, despite the actual presence of greenspaces in those areas. Either way, these findings suggest inequalities between formal and informal neighborhood in opportunities for recreation as manifested by self-reported park proximity and use.

The strength of this study is twofold. From a theoretical perspective, interactions between park proximity and neighborhood characteristics in relation to park use are intrinsic to socioecological models on which most studies in the field are based. Despite this, few prior studies examined such interactions, and hence the potential theoretical contribution of this study is in its third research questions on park proximity influences on park use in varying neighborhood conditions. From an empirical perspective, by focusing on Latin American cities, the current study addresses a research gap that was recently recognized by scholars (Sreetheran & Van Den Bosch, 2014) who called for more research on parks and crime in developing countries, where crime rates are generally high and greenspaces are scarce. Specifically, Latin American cities are underrepresented in research, despite their heightened vulnerability due to their greenspace deficit and inequalities (Rigolon et al., 2018), and high crime rates (Sreetheran & Van Den Bosch, 2014). These conditions no doubt make urban parks in Latin America especially important as a health-inducing community intervention. The health promoting potential of parks in Latin America is further reinforced by our findings, which are well aligned with other data from this world region linking between park proximity and physical activity (Gomez et al., 2010; Hallal et al., 2010; Jáuregui et al., 2016).

Despite these strengths, this analysis is limited in several ways. First, as a cross-sectional survey, this study can determine associations, but not causality. Secondly, the use of self-reported data is subject to biases, such as respondents' memory and/or social desirability. This may be especially critical for social disorder variables that may be under-reported in high crime areas due to respondents' fear of being viewed or caught as informants. Similarly, the lack of objectively measured built environment measures is another limitation, as these may not always be correlated with perceived measures. As a secondary analysis, the variables definitions are confined to the existing survey instrument, rather than designed to answer our research questions. For example, our main predictor variable, park proximity, is self-reported and does not include parks' internal characteristics, which may influence park use (McCormack et al., 2004). This concern may be alleviated to some degree given the high similarity between the CAF survey and the Neighborhood Environment Walkability Scale (NEWS), which has reported moderate validity (Adams et al., 2009) and when adapted to the Brazilian context has shown almost perfect test-retest reliability (de Matos Malavasi, da Silva Duarte, Both, & Reis, 2007). Another concern is that built environment and social disorder items are reported within a block from respondents' home, an area which may, or may not overlap with the nearest park. This is likely to affect results related to built-environment variables. However, for social disorder variables, this may be less of an issue given crime spillover effects, which may occur extending social disorder beyond parks into their surroundings (Crewe, 2001). Finally, our outcome variable generally defines park use per household, without specifying the park user, the means to access the park, the frequency of park visits, the activity in the park, or the park's location. While regular park use frequency was found to have adequate validity and reliability in a US sample (Evenson, Wen, Golinelli, et al., 2013; Evenson, Wen, Hillier, & Cohen, 2013), the measure in the current manuscript is blunter (yes/no) and its precise psychometric properties are unknown.

## 5 Conclusions

This study expands the existing literature by being the first in the region to use such a large and diverse sample. Our findings show a graded association between perceived park proximity and use with increased odds of park use associated with decreased walking time to the closest park. Additional factors associated with park use are neighborhood formality and related built environment characteristics, including paved streets and sidewalks. Park use was unrelated to most perceived neighborhood social disorder attributes, but there was an interaction with perceived indigence or begging, showing stronger association between perceived park proximity and use among those who reported higher prevalence of indigence or begging in their home neighborhood block.

Our findings overall highlight the importance of park proximity for park use in Latin American cities and underscore the role of neighborhood physical infrastructure (sidewalks, paved streets) in enhancing park use. The lack of observed associations between perceived social disorder and park use disconfirms the common notion, supported by theoretical and empirical literature, mostly from the Global North, that social disorder is a barrier to park use. Thereby our findings demonstrate how environmental and social influences on park use may vary across geographical and socio-cultural regions.

This study offers evidence-based policy implications for strategies to increase park use in Latin American cities. The findings strongly advocate for increased proximity between parks and residential areas. This can be done by equal distribution of parks within cities across different socioeconomic areas, including formal and informal neighborhoods. Proper pedestrian infrastructure, including paved streets and sidewalks, should be planned and maintained to connect residential areas with parks. Further investing in these routes (e.g., streetlights, urban design) may contribute to attract visitors. Strengthening initiatives that modify temporarily (or permanently) streets or land uses, such as pop up parks, Ciclovías (open streets) and play streets, can provide alternative, feasible and low-cost approaches to offer accessible recreational areas where no parks are available or distance to parks exceeds 20 min. Finally, our focus on Latin America and the findings identified are particularly important given the region's rapid growth and notable lack of green spaces. It provides initial evidence to compel policy makers and planners to consider green spaces as cities grow and are retrofitted.

## Funding

This project was supported by the 10.13039/100010269Wellcome Trust initiative, “Our Planet, Our Health” (Grant 205177/Z/16/Z).

## Declaration of competing interest

None.

## Figures and Tables

**Appendix 1:** Crude estimates^a^ of park use, based on logistic random intercept models (*N* = 7970).

	OR (CI)	*P*-value
Perceived park proximity (ref: more than 30 min)		
10–30 min walk	2.09 (1.89–2.32)***	.000
Less than 10 min walk	3.38 (3.01–4.10)***	.000

Neighborhood characteristics		
Neighborhood type		
Informal neighborhood	0.55 (0.48–0.64)***	.000
Built environment		
Perceived street lighting	1.13 (1.02–1.25)*	.018
Sidewalks	1.26 (1.12–1.43)***	.000
Paved street	1.45 (1.27–1.65)***	.000
Perceived high proximity to destinations	1.12 (1.01–1.24)*	.028

Perceived social disorder		
Drug use	0.91 (0.82–1.00)	.100
Gangs	1.00 (0.91–1.11)	.913
Prostitution	1.06 (0.93–1.21)	.368
Indigence or begging	1.11 (1.00–1.23)*	.036
Assault or crime	1.02 (0.92–1.12)	.736

^a^ All models are adjusted for the individual variables presented in Table 1: age, sex, length of neighborhood residency, having school aged children, automobile ownership, employment status (employed), education level (high-school or higher), number of persons per household, and self-rated health.

* *p* ≤ .05.

** *p* ≤ .01.

*** *p* ≤ .001.

**Appendix 2:** Descriptive statistics of the study sample stratified by city (n, %).

	BA (*n* = 1034)	LAP (*n* = 528)	SP (*n* = 628)	FOR (*n* = 938)	BOG (*n* = 1021)	QUI (*n* = 618)	LIM (*n* = 663)	MVD (*n* = 617)	CCS (*n* = 1043)	PAC (*n* = 318)	MEX (*n* = 562)
Park use (ref: non users)	720 (69.90%)	349 (67.50%)	342 (55.70%)	335 (35.71%)	730 (71.92%)	512 (82.85%)	483 (72.74%)	475 (76.74%)	465 (44.67%)	170 (53.46%)	436 (77.58%)

Park proximity											
Less than 10 min walk	719 (69.81%)	259 (50.10%)	217 (35.34%)	590 (62.90%)	535 (52.71%)	322 (52.10%)	440 (66.27%)	467 (75.44%)	396 (38.04%)	158 (49.69%)	251 (44.66%)
10–30 min walk	236 (22.91%)	204 (39.46%)	230 (37.46%)	218 (23.24%)	308 (30.34%)	215 (34.79%)	183 (27.56%)	119 (19.22%)	399 (38.33%)	98 (30.82%)	257 (45.73%)
More than 30 min	75 (7.28%)	54 (10.44%)	167 (27.20%)	130 (13.86%)	172 (16.95%)	81 (13.11%)	41 (6.17%)	33 (5.33%)	246 (23.63%)	62 (19.50%)	54 (9.61%)

Neighborhood type											
Informal neighborhood	419 (40.68%)	0 (0%)	0 (0%)	329 (35.07%)	425 (41.87%)	0 (0%)	0 (0%)	0 (0%)	378 (36.31%)	0 (0%)	0 (0%)

Built environment											
Good streetlights	406 (39.46%)	202 (39.61%)	433 (70.64%)	581 (62.54%)	519 (51.34%)	352 (57.33%)	421 (63.79%)	324 (52.68%)	396 (38.04%)	215 (67.82%)	222 (39.78%)
Sidewalks	808 (78.45%)	404 (78.14%)	593 (96.58%)	848 (90.41%)	559 (55.07%)	562 (90.94%)	548 (82.53%)	485 (78.35%)	449 (43.13%)	201 (63.21%)	124 (22.06%)
Paved street	762 (73.98%)	239 (46.23%)	592 (96.42%)	708 (75.48%)	609 (60%)	441 (71.36%)	537 (80.87%)	562 (90.79%)	970 (93.18%)	292 (91.82%)	533 (94.84%)
High access to non-park destinations	683 (66.31%)	159 (30.75%)	232 (37.79%)	585 (62.37%)	437 (43.05%)	274 (44.34%)	335 (50.45%)	338 (54.60%)	546 (52.45%)	119 (37.42%)	167 (29.72%)

Social disorder											
Drug use	695 (67.48%)	128 (24.76%)	386 (62.87%)	691 (73.67%)	600 (59.11%)	322 (52.10%)	394 (59.34%)	435 (70.27%)	541 (51.97%)	123 (38.68%)	283 (50.36%)
Gangs	514 (49.90%)	224 (43.33%)	148 (24.10%)	449 (47.87%)	503 (49.56%)	309 (50%)	335 (50.45%)	225 (36.35%)	480 (46.11%)	99 (31.13%)	233 (41.46%)
Prostitution	152 (14.76%)	54 (10.44%)	124 (20.20%)	352 (37.53%)	69 (6.80%)	35 (5.66%)	76 (11.45%)	114 (18.42%)	276 (26.51%)	47 (14.78%)	121 (21.53%)
homeless and panhandlers	456 (44.27%)	134 (25.92%)	311 (50.65%)	579 (61.73%)	446 (43.94%)	176 (28.48%)	216 (32.53%)	414 (66.88%)	502 (48.22%)	95 (29.87%)	214 (38.08%)
Assault or crime	584 (56.70%)	189 (36.56%)	302 (49.19%)	603 (64.29%)	468 (46.11%)	238 (38,51%)	330 (49.70%)	362 (58.48%)	536 (51.49%)	111 (34.91%)	236 (41.99%)

Sociodemographic characteristics											
Age	39.75 (11.24)	38.87 (10.33)	38.29 (10.69)	39.38 (10.94)	40.69 (11.46)	39.32 (11.23)	39.52 (10.86)	40.79 (11.48)	39.37 (11.38)	40.72 (11.37)	42.11 (9.91)
Female (ref: male)	585 (56.80%)	277 (53.58%)	539 (58.47%)	602 (64.18%)	675 (66.50%)	348 (56.31%)	382 (57.53%)	377 (60.90%)	570 (54.76%)	161 (50.63%)	317 (56.41%)
Having school aged children (ref: no)	748 (72.62%)	355 (68.67%)	402 (65.47%)	645 (68.76%)	611 (60.20%)	407 (65.86%)	456 (68.67%)	382 (61.71%)	658 (63.21%)	173 (54.40%)	342 (60.85%)
Automobile owner (ref: no)	380 (36.89%)	156 (30.17%)	302 (49.19%)	163 (17.38%)	259 (25.52%)	175 (28.32%)	121 (18.22%)	224 (36.19%)	225 (21.61%)	154 (48.43%)	230 (40.93%)
Employed (ref: unemployed)	683 (66.31%)	378 (73.11%)	385 (62.70%)	501 (53.41%)	620 (61.08%)	387 (62.62%)	412 (62.05%)	394 (63.65%)	688 (66.09%)	215 (67.62%)	315 (56.05%)
High school education or higher	438 (42.44%)	355 (68.67%)	337 (54.89%)	294 (31.34%)	581 (57.24%)	254 (41.10%)	520 (78.08%)	224 (36.19%)	663 (63.57%)	217 (68.24%)	247 (43.96%)

Self-rated health											
Bad	20 (1.94%)	24 (4.64%)	32 (5.21%)	77 (8.21%)	32 (3.15%)	27 (4.37%)	28 (4.22%)	19 (3.07%)	11 (1.06%)	7 (2.20)	23 (4.09%)
Regular	222 (21.55%)	317 (61.32%)	172 (28.01%)	349 (37.21%)	255 (25.12%)	236 (38.19%)	323 (48.64%)	165 (26.66%)	197 (18.92%)	106 (33.33%)	218 (38.79%)
Good	788 (76.50%)	176 (34.04%)	410 (66.78%)	512 (54.58%)	728 (71.1 = 72%)	355 (57.44%)	313 (47.14%)	435 (70.27%)	833 (80.02%)	205 (64.47%)	321 (57.12%)
Length of neighborhood residency	21.32 (14.52)	16.94 (13.93)	21.51 (14.79)	22.67 (13.89)	14.80 (12.14)	15.27 (12.80)	20.38 (14.15)	18.53 (16.57)	25.52 (15.79)	20.08 (14.92)	24.47 (15.89)
Num of person in the household	4.47 (1.92)	4.56 (1.70)	3.96 (1.61)	4.36 (1.61)	4.12 (1.58)	4.27 (1.62)	4.39 (1.64)	3.84 (1.76)	4.35 (1.75)	4.29 (1.83)	4.28 (1.45)

**Appendix 3:** Adjusted^a^ associations between park proximity and park use in sub-samples defined by neighborhood formality and individual sociodemographic characteristics (logistic random intercept models, N = 7970).

		Park proximity (ref: more than 30 min)	OR (CI)	P-value
Neighborhood type	Informal neighborhoods	10–30 min walk	1.58 (1.17–2.14)**	.003
		Less than 10 min walk	2.93 (2.16–3.95)***	.000
	Formal neighborhoods	10–30 min walk	2.23 (1.85–2.68)***	.000
		Less than 10 min walk	3.50 (2.93–4.20)***	.000
Sex	Female	10–30 min walk	3.30 (2.71–4.02)***	.000
		Less than 10 min walk	2.11 (1.73–2.58)***	.000
	Male	10–30 min walk	3.36 (2.62–4.31)***	.000
		Less than 10 min walk	1.97 (1.53–2.53)***	.000
Age	20–39	10–30 min walk	2.27 (1.83–2.84)***	.000
		Less than 10 min walk	3.71 (2.98–4.61)***	.000
	40–65	10–30 min walk	1.83 (1.46–2.29)***	.000
		Less than 10 min walk	3.00 (2.41–3.74)***	.000
Education	Less than high-school	10–30 min walk	3.37 (2.82–4.02)***	.000
		Less than 10 min walk	2.14 (1.78–2.56)***	.000
	High-school or higher	10–30 min walk	1.71 (1.24–2.38)**	.001
		Less than 10 min walk	3.18 (2.31–4.35)***	.000

^a^ All models are adjusted for the individual variables presented in Table 1: age, sex, length of neighborhood residency, having school aged children, automobile ownership, employment status (employed), education level (high-school or higher), number of persons per household, and self-rated health.

* p ≤ .05

** p ≤ .01

*** p ≤ .001

**Appendix 4:** City-specific models to predict park use by park proximity, built environment, socio-disorder and sociodemographic characteristics.

	BA	LAP	SP	FOR	BOG	QUI	LIM	MVD	CCS	PAC	MEX
Park proximity (ref: more than 30 min)											
Less than 10 min walk	**2.98** (1.73–5.14)***	1.53 (0.80–2.90)	**2.39** (1.55–3.70)***	**6.94** (3.92–12.28)***	**2.19** (1.45–3.31)***	1.24 (0.64–2.40)	**4.63** (2.26–9.52)***	**2.86** (1.28–6.41)*	**3.54** (2.37–5.29)***	**4.70** (2.37–9.31)***	**8.04** (3.75–17.24)***
10–30 min walk	1.27 (0.72–2.25)	1.64 (0.85–3.16)	**1.70** (1.11–2.60)*	1.75 (0.92–3.31)	**1.72** (1.12–2.64)*	1.43 (0.73–2.81)	**3.72** (1.75–7.89)***	1.84 (0.774.15-)	2.73(1.89–3.93)***	**3.62** (1.73–7.57)***	**3.05** (1.51–6.17)**

Neighborhood type											
Informal neighborhood	**0.50** (0.34–0.73)***	NI	NI	1.13 (0.81–1.58)	**0.46** (0.32–0.67)***	NI	NI	NI	**0.61** (0.45–0.83)**	NI	NI

Built environment											
Streetlights within three blocks	1.18 (0.81–1.72)	1.06 (0.70–1.59)	0.77 (0.52–1.16)	**0.67** (0.48–0.92)*	**1.55** (1.12–2.16)**	1.13 (0.70–1.83)	1.06 (0.72–1.58)	1.15 (0.75–1.78)	**0.61** (0.45–0.83)*	1.10 (0.43–1.90)	0.99 (0.59–1.68)
Sidewalks	1.06 (0.69–1.65)	1.35 (0.85–2.15)	**0.29** (0.09–0.97)*	**1.84** (1.03–3.30)*	**1.46** (1.04–2.05)*	**2.43** (1.14–5.19)*	1.32 (0.66–2.63)	1.21 (0.68–2.14)	**0.70** (0.52–0.96)*	1.15 (0.69–1.92)	**1.87** (1.02–3.44)*
Paved street	0.89 (0.58–1.34)	1.33 (0.89–2.00)	2.39 (0.78–7.30)	0.79 (0.54–1.16)	1.18 (0.83–1.68)	1.04 (0.60–1.80)	1.49 (0.76–2.90)	0.86 (0.38–1.97)	1.03 (0.60–1.75)	1.29 (0.53–3.12)	**2.94** **(1.18–7.30)**
High access to non-park destinations	1.01 (0.73–1.39)	1.09 (0.70–1.71)	0.81 (0.56–1.15)	**0.72** (0.53–0.99)*	0.78 (0.57–1.07)	**1.62** (1.00–2.64)*	1.27 (0.87–1.85)	0.88 (0.57–1.35)	1.15 (0.86–1.53)	1.35 (0.81–2.29)	0.82 (0.48–1.37)

Social disorder											
Drug use	**0.54** (0.37–0.79)**	1.46 (0.90–2.36)	1.05 (0.73–1.52)	1.18 (0.82–1.70)	0.72 (0.51–1.03)	0.74 (0.46–1.21)	**0.55** (0.36–0.83)***	1.05 (0.66–1.67)	1.31 (0.96–1.77)	0.78 (0.45–1.33)	1.09 (0.65–1.86)
homeless and panhandlers	**1.94** **(10.40–**2.68)***	0.97 (0.61–1.53)	1.27 (0.90–1.80)	1.08 (0.79–1.48)	**1.53** (1.10–2.13)*	1.21 (0.72–2.04)	0.93 (0.62–1.38)	1.01 (0.66–1.56)	0.85 (0.64–1.12)	1.24 (0.70–2.18)	0.91 (0.57–1.44)

Sociodemographic characteristics											
Age	**0.96** (0.94–0.97)***	0.99 (0.97–1.02)	**0.98** (0.96–0.99)*	**0.98** (0.96–0.99)**	**0.96** (0.95–0.98)***	0.99 (0.96–1.00)	0.99 (0.97–1.00)	**0.94** (0.92–0.97)***	0.99 (0.98–1.00)	0.99 (0.97–1.01)	0.99 (0.96–1.02)
Female (ref: male)	1.31 (0.94–1.82)	0.87 (0.56–1.36)	0.81 (0.56–1.17)	0.83 (0.61–1.15)	0.74 (0.52–1.05)	0.94 (0.57–1.54)	0.83 (0.53–1.29)	0.82 (0.53–1.25)	1.07 (0.80–1.43)	1.06 (0.61–1.82)	1.65 (0.94–2.89)
Having school aged children (ref: no)	**1.55** (1.09–2.20)*	1.09 (0.71–1.68)	**1.50** (1.04–2.17)*	0.94 (0.67–1.33)	0.98 (0.69–1.39)	**1.74** (1.08–2.81)*	**1.62** (1.08–2.42)*	1.16 (0.73–1.85)	1.12 (0.83–1.52)	1.09 (0.64–1.89)	1.38 (0.79–2.41)
Automobile owner (ref: no)	0.98 (0.71–1.36)	1.29 (0.81–1.99)	1.14 (0.81–1.63)	0.77 (0.51–1.17)	1.1.04 (.69–1.57)	1.36 (0.79–2.33)	1.00 (0.61–1.64)	0.90 (0.58–1.40)	0.88 (0.63–1.23)	**0.61** (0.37–0.99)*	1.47 (0.89–2.42)
Employed (ref: unemployed)	1.19 (0.84–1.67)	1.20 (0.75–1.93)	0.97 (0.67–1.40)	**1.46** (1.07–2.00)*	1.02 (0.73–1.40)	1.30 (0.80–2.12)	0.92 (0.60–1.43)	0.91 (0.59–1.42)	0.92 (0.68–1.23)	1.76 (0.98–3.16)	**1.85** (1.08–3.18)*
High school education or higher	0.89 (0.65–1.22)	1.82 (1.15–2.87)*	1.08 (0.76–1.56)	**1.59** (1.14–2.21)**	1.22 (0.86–1.71)	1.23 (0.75-2.02)	1.49 (0.95–2.34)	0.87 (0.50–1.49)	0.89 (0.66–1.20)	0.74 (0.42–1.29)	1.44 (0.89–2.35)
Self-rated health	1.04 (0.76–1.41)	**1.47** (1.01–2.13)*	1.25 (0.93–1.69)	1.22 (0.96–1.56)	0.99 (0.74–1.32)	1.44 (0.98–2.13)	1.23 (0.87–1.73)	1.36 (0.94–1.97)	1.34 (0.97–1.84)	1.45 (0.90–2.32)	1.25 (0.85–1.84)
Length of neighborhood residency	0.99 (0.99–1.006)	1.00 (0.99–1.01)	1.00 (0.99–1.02)	0.99 (0.98–1.00)	0.99 (0.98–1.00)	0.99 (0.98–1.01)	1.00 (0.99–1.02)	1.00 (0.99–1.02)	1.00 (0.99–1.01)	0.99 (0.97–1.00)	**0.98** (0.97–0.99)*
Num of person in the household	1.07 (0.98–1.16)	1.11 (0.98–1.26)	1.04 (0.93–1.16)	1.08 (0.98–1.19)	**1.17** (1.06–1.29)**	1.06 (0.92–1.22)	1.04 (0.93–1.17)	1.06 (0.93–1.21)	1.05 (0.98–1.14)	0.91 (0.79–1.05)	1.10 (0.94–1.29)
Constant	4.30 (0.99–18.71)	**0.15** (0.03–0.78)*	1.02 (0.19–5.43)	**0.12** (0.03–0.50)**	**4.62** (1.14–18.57)*	0.47 (0.08–2.91)	0.31 (0.06–1.66)	5.73 (0.81–40.37)	**0.20** (0.05–0.83)*	0.24 (0.03–2.20)	0.10 (0.01–1.05)
Number of observations	1034	528	628	938	1021	618	663	617	1043	318	562
AIC	1124.58	652.89	829.44	1093.24	1062.94	550.54	740.94	637.41	1368.35	428.74	541.71

* p ≤ .05

** p ≤ .01

*** p ≤ .001

Significant values are in bold.
